# Psychometric evaluation of the unmet needs instrument for carers of people with dementia (UNI-C)

**DOI:** 10.1186/s41687-025-00856-7

**Published:** 2025-03-11

**Authors:** Elise Mansfield, Emilie Cameron, Matthew Clapham, Alix Hall, Allison Boyes

**Affiliations:** 1 Health Behaviour Research Collaborative, School of Medicine and Public Health, College of Health, Medicine and Wellbeing, University of Newcastle, Callaghan, NSW Australia; 2 Equity in Health and Wellbeing Research Program, Hunter Medical Research Institute, New Lambton Heights, NSW Australia; 3 Clinical Research Design and Statistical Services, Hunter Medical Research Institute, New Lambton Heights, NSW Australia; 4 Population Health Research Program, Hunter New England Local Health District, Wallsend, NSW Australia

**Keywords:** Needs assessment, Support person, Caregiver, Alzheimer’s disease, Measurement, Psychometric evaluation

## Abstract

**Background:**

Carers play an important role in providing practical and emotional support to people with dementia. There is a need to ensure carers are supported in this role to maximise their ability to provide care while maintaining their own wellbeing. This study aimed to develop a psychometrically rigorous self-report instrument to assess the unmet needs of carers of people with dementia.

**Methods:**

The Unmet Needs Instrument for Carers of People with Dementia (UNI-C) was developed using a multi-methods approach including a comprehensive literature review, in-depth interviews and focus groups, expert opinion, cognitive interviews and pilot testing. A cross-sectional survey of 169 carers was used to examine the psychometric characteristics of the instrument. Specifically, internal reliability, and structural and convergent validity were assessed and the number of items reduced.

**Results:**

The original 80 item instrument was reduced to 46 items across four domains based on prevalence, item-total and pairwise correlations, factor loadings and clinical relevance. The resulting instrument, named the UNI-C46, showed acceptable evidence of structural and convergent validity, and good internal reliability (α = 0.94) and acceptability. The four domains measure ‘Your own wellbeing’ (16 items), ‘Finding information’ (12 items), ‘Managing practical needs’ (10 items), and ‘Managing dementia symptoms’ (8 items).

**Conclusions:**

This study provides the first demonstration of reliability and validity of the UNI-C46. Further testing of these properties with a larger and more representative sample of carers is recommended.

## Abstract (fr)

Carers play an important and ongoing role in supporting those with dementia living in the community. While caring can be rewarding, carers can also experience significant burden due to the caring role. Improving support for carers of people with dementia is a public health priority. Unmet needs assessment offers a practical approach to identifying areas in which carers require more support. We developed the Unmet Needs Instrument for Carers of People with Dementia (UNI-C) using a rigorous and accepted measure development process including literature reviews, expert opinion, interviews and focus groups with carers, pilot testing and psychometric evaluation. The final instrument measures carers unmet needs across four domains: Your own wellbeing, finding information, managing practical needs and managing dementia symptoms. The self-report instrument is suitable for use in clinical and research settings.

## Background

In Australia, approximately 67% of people with dementia are cared for at home by relatives or friends [1]. Carers play an important role in providing both practical and emotional support to people with dementia. While caregiving for a person with dementia can be a rewarding role, it often comes at a substantial personal cost, placing significant strain on the carer’s physical and mental health [1]. Given that dementia is a progressive illness with cognitive decline often occurring over a number of years, these burdens can become overwhelming, leading to carer burn out and placement of the person with dementia in institutional care [2, 3]. International action plans identify improving support for caregivers of people with dementia as a priority [4].

Unmet needs assessment offers a practical approach to identifying areas in which carers require more support. Unmet needs instruments ask about the extent to which individuals require additional support across different areas of their lives to improve their health and wellbeing [5]. Assessing unmet needs provides an opportunity to assess the gap between an individual’s concerns and the level of assistance they require. This approach is particularly useful in clinical contexts to identify individuals who are in need of the most support, as well as at a population level to identify needs that are frequently expressed, to assist in policy and care planning [6]. To ensure the outcomes of unmet needs assessment are useful for intervention development, the instrument should take a holistic approach and recognise the multi-faceted needs that are relevant to the carer’s wellbeing [7].

Instruments are currently available to assess the unmet needs of carers, however they have a number of limitations [8, 9]. Many rely on clinician or researcher assessments, including The Johns Hopkins Dementia Care Needs Assessment (JHDCNA) [10], care needs assessment pack for dementia (CarenapD) [11] and Caregiver Needs Assessment for Dementia (CNA-D) [12]. However, as research consistently demonstrates poor agreement between proxy rating and the individual’s rating of their own needs, these instruments may not adequately capture a carer’s own perceived needs [8, 13]. Some instruments designed primarily to identify needs of the person with dementia also include a small sub-set of items to assess carer unmet needs [14, 15], however, given the complexity of the caregiving experience, this approach is insufficient to adequately capture the multi-faceted nature of the caregiving experience and the scope of unmet needs that may arise [8, 9]. Psychometric characteristics of existing measures have also been poorly assessed [9].

To overcome the limitations of previous instruments, we developed a comprehensive self-report unmet needs instrument, the 80-item Unmet Needs Instrument for Carers of People with Dementia (UNI-C). An analysis of the prevalence and type of unmet needs experienced by carers of people with dementia using the 80-item UNI-C is reported elsewhere [16]. To increase feasibility and acceptability of this instrument in clinical and research settings, the current study aims to develop and evaluate the psychometric properties of a brief version of this instrument, the UNI-C46.

## Method

A multi-methods approach was used to develop an unmet needs instrument for carers of people with dementia. We used a reflective model using a classical test theory approach. Results are reported in line with Consensus-based Standards for the selection of health Measurement Instruments (COSMIN) [17] guidance. The process included the following steps: 1. Identification of target population; 2. Item generation; 3. Instrument development; 4. Administration of instrument; and 5. Psychometric evaluation of instrument. Psychometric characteristics of the instrument were evaluated with a secondary analysis of data collected from 169 participants.

### Target population

Carers were defined as people who have a significant personal relationship and are a main source of practical and emotional support to a person diagnosed with dementia by a medical professional, were aged at least 18 years, and were English speaking.

### Item generation

#### Literature review

A working group consisting of behavioural scientists (n = 5), a statistician (n = 1), health care professionals working in aged care (n = 2) and carers (n = 3) were responsible for generating items. The working group conducted a review of the peer-reviewed and grey literature on the needs, concerns or impacts experienced by carers of people with dementia, resulting in a list of 146 possible items.

#### In-depth interviews and focus groups

We conducted telephone interviews and a focus group with carers of people with dementia on the main issues they have needed help with across a broad range of areas (e.g. emotional, social, practical, financial, legal). Carers were recruited from two geriatricians’ clinics in regional NSW. Eligible carers were approached by the clinic nurse and invited to participate. All participants provided informed written consent. Carers were given the option to complete an interview with a researcher or participate in an in-person focus group moderated by a researcher. Using a semi-structured interview, carers were asked about their most recent needs, including whether there were any difficulties which occurred more frequently (e.g. on a daily basis). They were also asked about whether their needs had changed over time. Data saturation was reached after interviews with 18 carers and one focus group involving 3 carers. Interviews and the focus group were audio-recorded and the content analysed using the constant comparative method. Important themes and items were identified and used to refine the original item pool down to 77 items.

### Development of the UNI-C

**Fig. 1:**
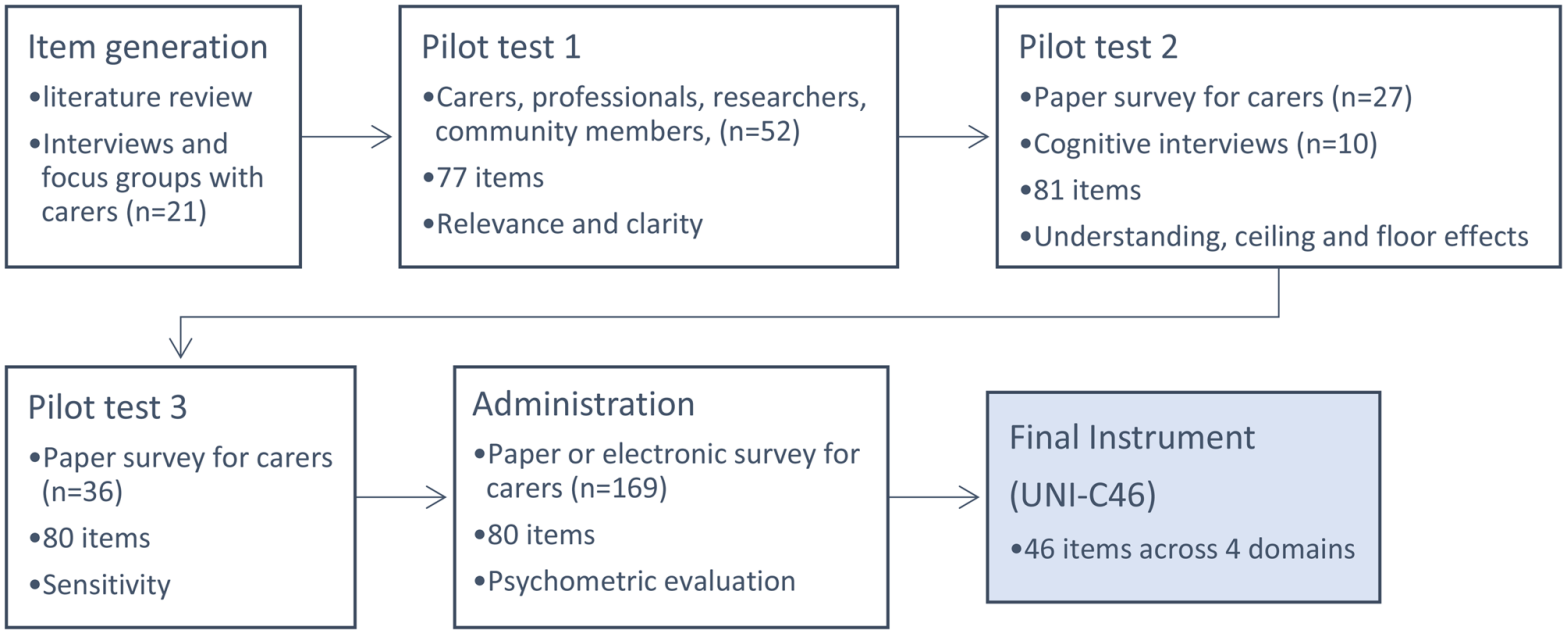
Flowchart of the development process from item generation to final version of UNI-C46

An iterative process was used where draft versions of the instrument were reviewed for relevance, clarity, redundancy and completeness and items were continuously refined until no further revisions were indicated. Three rounds of pilot testing were conducted, each with a unique sample of carers. The final UNI-C instrument was then administered to a new sample of carers. The process is outlined in Fig. 1.

#### First pilot test

The first draft instrument comprised 77 items divided into 9 sub-categories (practical, emotional, social, information, symptoms, advocacy, future care, financial, legal) with a response scale consisting of four options: This has not been a concern for me; This has been a concern but I don’t need help with this; This has been a concern but I got the help that I needed; This has been a concern and I need help with this. The first draft of the unmet needs instrument was sent to carers (n = 14), health care providers (n = 10), behavioural scientists (n = 10), managers of community aged care services (n = 3), staff of relevant community services (n = 4), community members (n = 10), and a legal professional (n = 1). Respondents were asked to provide feedback on: i) whether any items could be removed or added; ii) whether instructions, items and response options were easy to understand; and iii) relevance and impact of items for carers of people with dementia. Items identified by the majority of respondents as being redundant were eliminated, and some additional items identified as missing were added. The resulting second draft of the instrument had 81 items.

#### Second pilot test

To examine response rate and ceiling and floor effects (where all respondents gave the same response for an item), the second draft of the instrument was mailed from one geriatrician’s clinic in regional NSW to 50 carers of people with dementia. The clinic administrative assistant extracted a list of eligible carers and telephoned each carer until 50 consented to receive the instrument. Non-responders received one reminder letter including a second copy of the instrument. Of the 50 eligible carers, 27 (54%) returned a completed instrument. The rate of item completion was high, with 3.2% missing data across all needs items. Three items had a poor spread of responses with no participant indicating it was a need and were removed. No item was endorsed as a need by all respondents.

Cognitive interviews were conducted with an additional 10 carers with a variety of individual and social characteristics recruited through a respite centre, to ensure the relevance, comprehensiveness and comprehensibility of the response scale and items. Carers were asked to ‘think-aloud’ as they completed the instrument and share what they believed each item was asking, and the reason for selecting a response. This process resulted in changes being made to increase the clarity of the response options, and item wording to ensure a grade 5–7 reading level. Based on additional feedback provided by respondents, two items related to future care and understanding the situation were added.

The resulting third version of the instrument comprised 80 items and response options: “I don’t need help”; “I get help”; and “I need more help”.

#### Third pilot test

The third draft version of the instrument was sent to an additional 64 carers recruited through an audit of clients attending a geriatrician clinic and respite centre for people with dementia. 36 returned the completed instrument (response rate 56%). There was 6.0% missing data across needs items. No items were identified as being redundant, and no further items were identified as being relevant for addition to the instrument. The response scale was changed from a three point to a four-point scale with “I need more help” split into “I could have used a little more help”, and “I could have used a lot more help” to allow participants to indicate relevance and importance of the need.

#### UNI-C instrument

The resulting unmet needs instrument for carers of people with dementia (UNI-C) consisted of 80 items grouped into seven domains [16].

### Administration of instrument

#### Procedure

Study eligibility, recruitment and data collection procedures are described in detail elsewhere [16]. Briefly, a cross-sectional survey of carers was conducted. Eligible carers identified through 10 geriatrician clinics, three respite centres for people with dementia, six carer support groups, 34 community groups and two aged care providers located in regional areas of NSW and Victoria were sent a questionnaire package including a cover letter, information sheet, survey and reply-paid envelope. Return of a completed survey was taken as implied consent to participate. Participants could complete the questionnaire on paper or online. Non-responders were sent a reminder letter including a second copy of the survey after 4 weeks. Recruitment was conducted from July 2018 to June 2020.

#### Measures

The self-report questionnaire comprised the following:

- *Carer unmet needs:* assessed by the UNI-C.
- *Depression:* assessed using the Patient Health Questionnaire-9 (PHQ-9) [18], a self-administered measure of depression severity with adequate reliability and evidence of validity. Respondents indicate how frequently they have experienced each symptom over the past 2 weeks on a four-point Likert scale (Not at all/Several days/More than half the days/Every day). Total scores range from 0 to 27, with a score of at least 10 indicative of moderate to severe depression, with 88% sensitivity and 88% specificity [18].
- *Health related Quality of Life:* assessed using the Medical Outcomes Study Short Form 12 Version 2 (SF-12 v2) [19], which assesses health and wellbeing across eight domains: general health, physical functioning, role functioning (physical), bodily pain, vitality, role functioning (emotional), mental health, and social functioning. Physical component summary (PCS) and mental component summary (MCS) scores are calculated, with each score ranging from 0 to 100. Higher scores indicate better physical and mental health. The scale demonstrates sufficient internal consistency, and evidence of discriminant, convergent and predictive validity [20].
- *Carer sociodemographic characteristics:* including sex, age, postcode, marital status, employment status, highest level of education, Aboriginal or Torres Strait Islander status, private health insurance status, concession card status, other health conditions, number of years caring for the person with dementia, number of hours per week spent providing care, relationship with the person with dementia, and living arrangements.
- *Sociodemographic and disease characteristics of the person with dementia:* Carers were asked about characteristics of the person they support, including their sex, age, Aboriginal or Torres Strait Islander status, month and year of diagnosis, dementia sub-type (if known), other chronic conditions, and perception of the severity of dementia symptoms (on a scale of 1–10).
- *Questionnaire acceptability*: Participants were asked to record the approximate length of time it took to complete the full questionnaire, whether they found the items easy to understand, and whether they found any items distressing.

### Development and psychometric evaluation of the UNI-C46

A Classical Test Theory approach was used to develop the UNI-C46 and examine its psychometric properties including reliability (internal consistency), validity (structural and convergent) and acceptability [21]. Statistical analyses were programmed using R version 4.1.1 [22]. Missing items were replaced with “I didn’t need help”. If all items on this measure were missing the participant was removed from the analysis (n = 3).

#### Item reduction

We explored whether it was possible to reduce the UNI-C to a sub-set of items that were most relevant to carers and had high utility in clinical settings. The original 80 items were submitted to an initial exploratory principal components factor analysis with polychoric correlation used in the factor extraction. We used principal component analysis to reduce the dimensionality of our dataset while organising the variables into coherent domains. This allowed us to make a more concise representation of the underlying patterns in the items. Scree plots and parallel analysis elbow method indicated that seven domains were acceptable. Item-total and inter-item correlations were performed. Criteria for item removal were: less than 10% of sample reported an unmet need, item-total correlations < 0.3, inter-item correlations > 0.8 (indicating redundancy), or factor loadings less than 0.4. Some items that met the criteria for removal were retained due to perceived clinical relevance.

#### Structural validity

Exploratory principal components factor analysis was used to assess dimensionality of the reduced set of items. Polychoric correlation was used in the factor extraction. Scree plots and parallel analysis with eigenvalues were calculated to inform the number of factors to extract and Oblimin rotated factor loadings were calculated. Items were included in factors using the maximum absolute factor loading. The final factor structure was selected based on scree plots and parallel analysis, factor loadings > 0.3, few cross-loadings of items, and no factors comprising fewer than 3 items [23]. The chosen solution also loaded onto theoretically plausible factors.

#### Internal reliability

Cronbach’s alpha coefficients were used to determine internal reliability of the instrument overall and by domain. An alpha coefficient of > 0.7 was considered acceptable [24, 25].

#### Convergent validity

Separate Pearson’s correlations were calculated between total number of unmet needs and: (i) scores on the PHQ-9, (ii) PCS and (iii) MCS scores on the SF-12 Version 2 (SF-12 v2). Correlations were considered to be moderate if they were between *r* = 0.3–0.7, and strong if greater than *r* = 0.7 [26]. Based on the results of previous studies, we expected moderate positive correlations between total number of unmet needs and PHQ-9 scores [27, 28], and moderate negative correlations with PCS and MCS scores on the SF-12 v2 [29].

## Results

### Sample characteristics

There were 414 eligible carers who were invited to participate. Of these, 196 completed and returned the questionnaire (response rate 47%). Of those who returned a questionnaire, 27 were excluded as they were ineligible (n = 26) or did not complete the UNI-C (n = 1), leaving 169 eligible carers for the analysis. A detailed breakdown of the demographic characteristics of the sample is reported elsewhere [16]. Briefly, the sample comprised mostly of females (74%, n = 123), and partners of the person with dementia (83%, n = 139), with a mean age of 71.8 years. Most (84%, n = 139) lived in major cities, and 61% (n = 101) lived in areas classified as ‘Not disadvantaged’ according to the SEIFA Index of Advantage and Disadvantage. Of the people with dementia who were receiving support from the carers in this sample, two-thirds were male (64%, n = 107), and had a mean age of 79.4 years (SD = 8.6). Carers reported a mean symptom severity for people with dementia of 5.9 (SD = 1.8) out of a possible 10. Compared to national demographic data on carers of people with dementia [30], our sample included a similar gender distribution (*p* = 0.635), but were older (*p* < 0.001) and included more carers who were a partner of the person with dementia (*p* < 0.001). The majority of carers (93%, n = 158) completed the pen and paper version of the survey.

### Item reduction

Of the original 80 UNI-C items, 34 were removed to produce the final UNI-C46 instrument. Eleven items were removed due to low prevalence (all those < 10%). Four items were removed due to low item-total correlation (<0.3), while one was retained (“Accessing financial assistance”) due to its high prevalence and impact. One item (“Getting enough sleep”) with a factor loading less than 0.4 was retained due to clinical relevance. For items with pairwise correlations above 0.75 the pair was reviewed and either 1 retained or both, resulting in 15 items being removed. This included 1 from each pair with a correlation above 0.9. All remaining items were then reviewed and a further 4 removed for being redundant.

### Structural validity

There were 11 factors with eigenvalues greater than 1. The scree plot indicated 3–5 factors should be retained. Parallel analysis using randomised data indicated the number of factors to retain was 3–7. Rotated factor loadings are provided for the 46 items with a four-factor solution (see Table 1). Factor loadings ranged from 0.32 to 0.9. This solution explained 55% of the variance. While solutions with more factors explained a higher proportion of the variance, this model was selected given the items loaded onto theoretically plausible factors.

**Table 1:** Rotated factor loadings for the four factor solution for the UNI-C46

Factor	Item	Loading
Your own wellbeing	Looking after your own health	0.66
	Getting enough sleep	0.46
	Balancing your role as a carer with other responsibilities (e.g., family, work)	0.60
	Managing stress	0.78
	Keeping up your social activities or hobbies	0.63
	Finding the emotional energy that you need to care for the person you support	0.90
	Worrying about what will happen in the future	0.71
	Feeling sad	0.82
	Feeling guilty if you enjoy yourself without the person you support	0.71
	Feeling unsure in your abilities as a carer	0.67
	Coping with changes in your relationship with the person you support	0.57
	Understanding the perspective of the person you support	0.49
	Explaining to others what you are going through	0.41
	Dealing with negative reactions of people towards the person you support	0.36
	Coping with thinking for the person you support as well as yourself	0.48
	Asking for help from family and friends	0.32
Finding information	Accessing a home care package with enough help for the person you support	0.38
	Accessing respite care (care that you or the person you support can stay in for a short time)	0.42
	Knowing what care providers to contact for the person you support and when	0.51
	Navigating the My Aged Care website	0.51
	Uncertainty about who will care for the person you support if something happens to you	0.40
	Finding support groups for carers	0.75
	Understanding how the dementia might change over time	0.75
	Finding information on possible treatments for dementia	0.86
	Being aware of clinical trials and research that we could be involved in	0.75
	Knowing when the person you support should move to a nursing home	0.67
	Accessing financial assistance	0.74
	Knowing what to do when the person you support can no longer make decisions on their own	0.58
Managing practical needs	Being at appointments with the person you support	0.46
	Helping the person you support with everyday tasks (e.g., bathing or dressing)	0.66
	Doing tasks which the person you support used to do (e.g., mowing the lawn, doing the laundry)	0.59
	Preparing meals that the person you support will eat	0.71
	Keeping the person you support in touch with family and friends	0.79
	Finding meaningful and enjoyable activities for the person you support	0.66
	Ensuring the person you support is safe	0.62
	Keeping the person you support living at home for as long as possible	0.64
	Encouraging the person you support to be more independent	0.76
	Involving the person you support in making decisions about their care	0.33
Managing dementia symptoms	The person you support having trouble remembering things	0.51
	Changes in mood of the person you support	0.55
	Sleep problems in the person you support	0.55
	Toileting issues in the person you support (e.g., constipation, getting to the toilet in time)	0.50
	Aggression from the person you support	0.67
	The person you support asking the same questions over and over	0.42
	The person you support following you around	0.41
	The person you support having a lack of understanding about the situation	0.46

Given the results of this analysis, the original seven hypothesised domains of the UNI-C were reduced to a four domain structure for the UNI-C46. The first factor, ‘Your own wellbeing’, accounted for 18% of the variance and comprised 16 items covering the carers own health and wellbeing concerns, such as “Feeling sad”, “Managing stress”, “Keeping up your social activities and hobbies” and “Coping with changes in your relationship with the person you support.” The second factor, ‘Finding information’, accounted for 15% of the variance and comprised 12 items covering difficulties with accessing information or support, such as “Understanding how dementia might change over time”, “Knowing what care providers to contact for the person you support and when”, and “Accessing financial assistance”. The third factor, ‘Managing practical needs’, accounted for 13% of the variance and included 10 items covering provision of support with day-to-day activities, such as “Helping the person you support with everyday tasks (e.g. bathing or dressing)”, “Finding meaningful and enjoyable activities for the person you support” and “Ensuring the person you support is safe”. The final factor, ‘Managing dementia symptoms’, comprised 8 items including “The person you support having trouble remembering things”, “Changes in the mood of the person you support” and “Aggression from the person you support”. It accounted for 9% of the variance.

Two items had similar factor loadings in two factors and were placed in the lower factor due to it being a better fit considering the other items comprising that factor. One of these items was “Asking for help from family and friends”, which originally loaded onto the ‘Managing practical needs’ factor (0.36) and was moved to the ‘Your own wellbeing’ factor (0.32). The other was “Accessing respite care” which was moved from ‘Managing dementia symptoms’ (0.43) to ‘Finding information’ (0.42).

### Internal reliability

The UNI-C46 showed good internal reliability overall (α = 0.94) and by domain: ‘Your own wellbeing’ (α = 0.89), ‘Finding information’ (α = 0.83), ‘Managing practical needs’ (α = 0.84), and ‘Managing dementia symptoms’ (α = 0.83).

### Convergent validity

The total number of unmet needs showed a moderate positive correlation with scores on the PHQ-9 (*r* = 0.58, *p* < 0.001), and a moderate negative correlation with the SF12 v2 MCS score (*r* = − 0.62, *p* < 0.001). There was no significant correlation between total unmet needs score and the SF12 v2 PCS score (*r* = − 0.01, *p* = 0.938).

### Acceptability

The full questionnaire took participants an average of 34 minutes (SD = 15 minutes) to complete. Over 90% of participants found the questions easy to understand (n = 128; 92%) and did not find any of the questions distressing (n = 139, 98%).

## Discussion

Comprehensive and robust assessment of unmet needs of carers of people with dementia can provide valuable data to shape service provision and decision making regarding allocation of resources to best meet carers’ needs. Effectively meeting carers’ needs may assist them to more effectively cope with the demands of the role. We aimed to provide a comprehensive, psychometrically robust and feasible self-report instrument that is useful in both research and practice settings to reliably identify the needs of carers of people with dementia. From a practical perspective, the instrument can be used to pinpoint the additional support or resources that are required to support carers more effectively. For example, a carer who indicated they were struggling with emotional fatigue by endorsing the item “Finding the emotional energy that you need to care for the person you support” (‘Your own wellbeing’ domain) may benefit from a referral to counselling services or support groups.

The UNI-C46 overcomes several limitations of previous dementia carer unmet needs instruments. It is a self-report rather than clinician or researcher-rated instrument, ensuring the views of carers themselves are accurately captured. It includes a graded rather than dichotomous response scale, allowing carers the opportunity to indicate both the existence of a need and the extent to which they require help with an identified need. This approach may be particularly useful when determining resource allocation to programs to address carer needs.

We aimed to develop an instrument that was as comprehensive as possible while also being feasible and acceptable for use in both clinical and research settings. We used a thorough and rigorous measure development and evaluation process including literature reviews, in-depth interviews and focus groups, and iterative rounds of feedback from carers, health and aged care providers and behavioural scientists to develop the original 80-item UNI-C. This process ensured the development of a comprehensive set of items for inclusion. The instrument was shortened to increase acceptability of administration using strict criteria to assess item relevance and redundancy. The final UNI-C46 had a four-factor structure that explained 55% of the variance and showed good internal reliability overall and by domain. The UNI-C46 also showed acceptable convergent validity, evidenced by moderate positive correlations with depression scores on the PHQ-9, and moderate negative correlations with health-related quality of life scores on the SF12 v2 MCS. While we did not assess completion time specifically for the UNI-C46, the average completion time for the full questionnaire including the 80-item version of the UNI-C was 34 mins, which is still substantially less than for other unmet needs measures [9].

The Dementia Carer Assessment of Support Needs Tool (DeCANT) is another self-report instrument to assess dementia carer needs that was published subsequent to the development of the UNI-C46 [31]. Similar to the UNI-C46, this measure uses a graded response scale to assess the extent of needs. The DeCANT showed a moderate fit for a four factor model comprising daily life, communication, focusing on themselves and maintaining their own wellbeing. Internal consistency reliability for the sub-scales was acceptable (α = 0.73–0.84). With some differences in wording and intent the 25 items of the DeCANT can largely be mapped to the UNI-C46. Additional items included in the UNI-C46 are mainly in the domains ‘Managing dementia symptoms’ (e.g. sleep and mood-related symptoms), and ‘Managing practical needs’ (e.g. keeping the person with dementia safe and maintaining the individual’s independence). While the DeCANT asks carers to think about their current needs, the UNI-C46 asks carers to report their needs over the past month, which may offer greater sensitivity in picking up needs that may recur in future. It appears both measures are promising and feasible tools for assessing unmet needs in this population. However, both require additional psychometric testing prior to recommending their widespread use. Further testing of their feasibility and acceptability in clinical settings is also warranted.

### Limitations and future directions

The results of this analysis should be considered in light of several limitations. Carers were recruited from a range of sources, however all were in contact with supports or geriatricians. Therefore, those not in connection with services and potentially with greater needs may have been missed. The sample size was relatively small for a psychometric analysis. While a general rule is to have 5 subjects per item (our study had 2.1), it is now accepted that components with 4 or more factor loadings above 0.6 are reliable regardless of sample size [32]. Our final structure had 3 of the 4 factors with at least 6 factor loadings greater than 0.6. The sample also included overrepresentation of older adults and carers who were a partner of the person with dementia relative to the general population. We recommend that the analysis be replicated with a larger more representative sample of carers of people with dementia, to ensure generalisability. This would also allow for an examination of psychometric properties across sub-groups of dementia carers (e.g. those who are caring for individuals with early vs late stage dementia), and carers of people with different sub-types of dementia (e.g. Alzheimer’s Disease, fronto-temporal dementia).

Our analysis of psychometric properties did not include assessment of test-retest reliability, responsiveness, or predictive validity. We were also unable to examine whether psychometric properties differed by survey administration mode, as so few participants completed the survey online. These properties could be examined in future longitudinal studies of unmet needs of carers using this measure. Completion time for the UNI-C46 should be assessed to increase confidence in its acceptability to respondents and utility in clinical settings.

## Conclusions

The UNI-C46 was developed and tested using a rigorous and accepted measure development process and was found to have acceptable structural and convergent validity, and good internal reliability. Further testing of these properties is needed with a larger and more representative sample of carers, including more extensive testing of reliability to ensure that scores are both stable as well as responsive to changes in unmet needs over time.

## Data Availability

The data that support the findings of this study are available from the corresponding author, [EM], upon reasonable request.

## List of abbreviations

CarenapD: Care needs assessment pack for dementia
CNA-D: Caregiver Needs Assessment for Dementia
COSMIN: Consensus-based Standards for the selection of health Measurement Instruments
DeCANT: Dementia Carer Assessment of Support Needs Tool
JHDCNA: Johns Hopkins Dementia Care Needs Assessment
MCS: Mental component of SF-12 summary
PCS: Physical component of SF-12 summary
PHQ-9: Patient Health Questionnaire-9
SF-12 v2: Medical Outcomes Study Short Form 12 Version 2
UNI-C: Unmet Needs Instrument for Carers of People with Dementia
